# Coordinated Translocation of Mammalian Gli Proteins and Suppressor of Fused to the Primary Cilium

**DOI:** 10.1371/journal.pone.0015900

**Published:** 2010-12-29

**Authors:** Huiqing Zeng, Jinping Jia, Aimin Liu

**Affiliations:** 1 Department of Biology, Eberly College of Science, The Pennsylvania State University, University Park, Pennsylvania, United States of America; 2 Center for Cellular Dynamics, Huck Institute of Life Sciences, The Pennsylvania State University, University Park, Pennsylvania, United States of America; Institute of Biophysics, Universidade Federal do Rio de Janeiro, Brazil

## Abstract

Intracellular transduction of Hedgehog (Hh) signals in mammals requires functional primary cilia. The Hh signaling effectors, the Gli family of transcription factors, and their negative regulator, Suppressor of Fused (Sufu), accumulate at the tips of cilia; however, the molecular mechanism regulating this localization remains elusive. In the current study, we show that the ciliary localization of mammalian Gli proteins depends on both their N-terminal domains and a central region lying C-terminal to the zinc-finger DNA-binding domains. Invertebrate Gli homologs Ci and Tra1, when over-expressed in ciliated mouse fibroblasts, fail to localize to the cilia, suggesting the lack of a vertebrate-specific structural feature required for ciliary localization. We further show that activation of protein kinase A (PKA) efficiently inhibits ciliary localization of Gli2 and Gli3, but only moderately affects the ciliary localization of Gli1. Interestingly, variants of Gli2 mimicking the phosphorylated or non-phosphorylated states of Gli2 are both localized to the cilia, and their ciliary localizations are subjected to the inhibitory effect of PKA activation, suggesting a likely indirect mechanism underlying the roles of PKA in Gli ciliary localization. Finally, we show that ciliary localization of Sufu is dependent on ciliary-localized Gli proteins, and is inhibited by PKA activation, suggesting a coordinated mechanism for the ciliary translocation of Sufu and Gli proteins.

## Introduction

Hedgehog (Hh) family of secreted proteins play pivotal roles in development, adult stem cell maintenance and cancers [1]. In *Drosophila*, Hh elicits transcriptional responses in target cells through a signal transduction pathway comprising its receptor Patched (Ptc), a serpentine receptor-like protein Smoothened (Smo), and a Hh signaling complex comprising a Fused kinase (Fu), a kinesin-like Costal2 (Cos2) and a transcription factor Cubitus interruptus (Ci). Ci is a dual-functional transcription factor, which, in the absence of Hh, is proteolytically processed into a transcriptional repressor. In the presence of Hh, full-length Ci is converted into a transcriptional activator that mediates the transcriptional responses of Hh target cells.

In mammals there is conservation of the roles of most Hh pathway regulators, such as Ptch1, Smo, Kif7 (Cos2 homologue) and Gli proteins (Gli1, 2 and 3, Ci homologue) [1], [2]. However, some aspects of Hh signal transduction are strikingly divergent. The primary cilium, a surface organelle that is not present in most *Drosophila* cells, plays an essential role in mammalian Hh signaling [3]. Detailed genetic analyses suggest that both the transcriptional activator and repressor functions of Gli proteins are compromised in mutant mouse embryos with defective cilia [4], [5], [6], [7]. However, whether cilia are essential for the activation of all three Gli proteins remains controversial because over-expression of Gli proteins, especially Gli1, is able to activate a Hh-responsive reporter gene in cultured cells independent of cilia [4], [8], [9].

In mammals, Smo is localized to the cilia in the presence of Hh and this localization is required, but not sufficient for the activation of a downstream response to Hh [10], [11], [12], [13]. Ptch1, which is localized to the cilia only when Hh is absent, appears to play an important role in regulating Smo localization [14]. All three mouse Gli proteins are also localized to the cilia in response to Hh, but the molecular mechanism underlying this localization and its importance in Gli activation have not been fully addressed [4], [15], [16].

Suppressor of Fused (Sufu) plays a negative role in Hh signal transduction in both *Drosophila* and mammals, but is essential for development only in mammals [17], [18], [19]. Sufu physically interacts with Ci/Gli proteins and at least part of its function is to sequester Ci/Gli proteins in the cytoplasm [20], [21], [22], [23], [24], [25]. In the presence of Hh, Sufu remains associated with Ci and enters the nuclei with Ci [26]. Additional evidence showed that Sufu directly influences the transcriptional activity of Gli proteins in the nucleus by recruiting histone deacetylation complex (HDAC) [27], [28]. However, this nuclear role of Sufu has been challenged in two recent studies [9], [29].

Consistent with the biochemical data showing direct physical interaction between Gli and Sufu, Sufu is also localized to the tips of cilia [4]. Importantly, Sufu remains associated with, and represses the activities of, Gli proteins in the absence of cilia [8], [9]. These studies suggest that the association between Sufu and Gli proteins does not require cilia. It is plausible that Sufu and Gli proteins are assembled into a protein complex prior to their localization to cilia, but this possibility has not been experimentally tested.

In the current study, we found that both the N-terminal region and a central region adjacent to the DNA binding zinc finger domain mediate ciliary localization of Gli2. Of interest, this central region is required for the ciliary localization of all three Gli proteins, suggesting a conserved mechanism for their ciliary trafficking. Invertebrate Gli homologues, such as Ci and Tra1, are not localized to the cilia when introduced into ciliated mammalian cells. We further show that activation of PKA prevents ciliary localization of Gli2 and Gli3, and to a lesser extent, Gli1. This effect of PKA is not through direct phosphorylation of the four serine residues in Gli2 that are critical for Gli2 processing and degradation. Finally, we show that the ciliary localization of Sufu is dependent on its association with Gli proteins, and is similarly prevented by PKA activation, providing direct evidence that these proteins are likely to be localized to the cilia as preassembled complexes.

## Materials and Methods

### Ethics Statement

All animal work conducted in this report is in accordance of national and international guidelines and was approved by IACUC (#29195 and #29214) at Penn State University.

### Mice

*Gli2^lacKI^, Gli3^Xt-J^* and *Sufu* mutants are kept on a 129S2/SvPasCrl background and genotyped as reported [17], [30], [31].

### DNA Constructs

Human Gli1, Gli3, and mouse Gli2, as well as mouse Sufu cDNAs (gifts of R. Toftgard and B. Wang), *Drosophila* Ci cDNA (gift of T. Holmgren) and *C. elegans* Tra1 cDNA (gift of D. Zarkower) were cloned into pEGFPC expression vectors (Clontech). Gli2P1-4, Gli2G2-4 and Gli2C1-4, into which Serine-to-Alanine mutations were introduced at target sites for PKA, GSK3 and CK1, were kindly provided by B. Wang and cloned into pEGFPC vectors. Truncated variants of Gli1, Gli2, Gli3, as well as Gli2SD1-4 in which Serine-to-Aspartic Acid mutations were introduced at target sites for PKA, were generated by a combination of restriction digestion and PCR strategies. The proper expression of all constructs was confirmed through immunoblot analyses (Fig. S1, S2, ).

### Immunoblot and immunoprecipitation

Immunoblot and immunoprecipitation analyses were performed according to previously published protocols [8]. Antibodies used in this study are: GFP (Invitrogen, A11122), Sufu (Santa Cruz Biotech, sc-28847), FLAG (Sigma, F3165).

### Cell culture and Immunocytochemistry

The establishment, transfection, cilia induction and immunocytochemistry analyses of mouse embryonic fibroblast culture (MEFs) were performed according to a previously published protocol [32]. Specifically, MEF cultures were established from whole E10.5 *Gli2^−/−^;Gli3^−/−^* mutant, E9.5 *Sufu^−/−^* mutant and wild type littermate embryos and were immortalized by stably expressing SV40 Large T Antigen (gift of B. Wang). For immunocytochemistry analyses, cells were transfected with DNA constructs expressing GFP-tagged proteins and cultured in medium containing 0.5% fetal bovine serum for 48 hours to allow ciliogenesis. Cells were then processed for immunofluorescence with antibodies against GFP and acetylated a-tubulin as a marker for the cilia. Ciliated cells with obvious GFP signal in cytoplasm or nucleus were scored for ciliary localization of the GFP-tagged proteins. For the localization of endogenous Sufu proteins in *Gli* mutant cells expressing various GFP-tagged Gli protein variants, ciliated cells with GFP fluorescent signals were scored. At least two independent experiments were performed for each protein. To activate PKA activity, cells are treated with 40 µM forskolin (CalBiochem, 344270) for 4 hours or 18 hours prior to fixation. 20 µM MG-132 (CalBiochem, 474790) was added to cells 4 hours prior to fixation to inhibit proteasome-mediated protein degradation.

## Results

### The N-terminus of the Gli2 protein plays an important but not essential role in its ciliary trafficking

**Figure 1:**
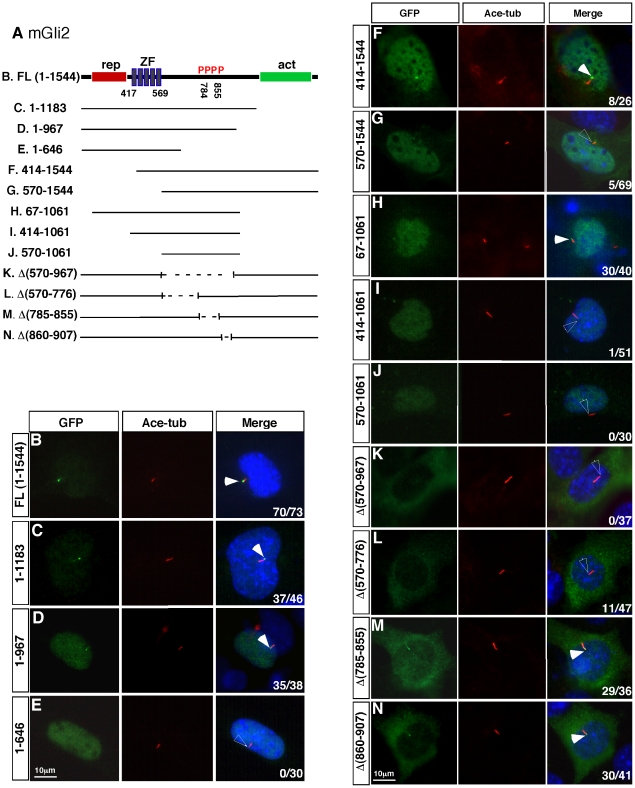
A central domain of Gli2 is essential for its ciliary localization. (A) Mouse Gli2 is composed of an N-terminal region (1–416), five zinc-finger motifs (ZF; 417–569) and a C-terminal region (570–1544). Transcriptional repressor (rep: red box) and activator (act: green box) activities are found in the N- and C-terminal regions, respectively. Four clusters of phosphorylation (P) target sites are located between residues 785–855. Schematics are shown for the deletions made in the Gli2 variants used in B–N. (B–N) Immunofluorescent images of MEFs transfected with GFP-tagged Gli2 variants are shown. Cilia are labeled with acetylated tubulin and nuclei are stained with DAPI. In the merged images, filled arrowheads indicate GFP-Gli2 at the tips of the cilia, unfilled arrowheads indicate the tips of cilia without GFP-Gli2 accumulation. Numbers at the lower-right corners of each image indicate numbers of cells with ciliary localization of GFP-tagged proteins over total numbers of transfected cells.

Mouse Gli2 protein is a bipartite transcription factor with a repressor domain at its N-terminus (residues 1 to 416), followed by a DNA binding domain comprising five zinc fingers (residues 417–569), and a C-terminally located activator domain (residues 570–1544) (Fig. 1A) [33]. By generating a series of C-terminally truncated Gli2 proteins, we found that more than half of the C-terminal region (968–1544) is not required for the ciliary localization of Gli2 (Fig. 1B–D; Table 1). Further truncation of the C-terminus completely abolishes the ciliary-localization of Gli2, suggesting that this region (647–967) constitutes at least part of the domain that mediates the ciliary localization of Gli2 (Fig. 1E; Table 1).

**Table 1:** The subcellular localization of various Gli proteins over-expressed in mouse ciliated fibroblasts.

Over-expressed proteins	Cells with ciliary localization of the protein of interest/transfected ciliated cells*	Localization of the protein of interest outside the cilia
GFP-mGli2		
1–1544	70/73	cytoplasm
1–1183	37/46	cytoplasm
1–967	35/38	nucleus
1–646	0/30	nucleus
414–1544	8/26	nucleus
570–1544	5/69	nucleus
67–1061	30/40	nucleus
414–1061	1/51	nucleus
570–1061	0/30	nucleus
Δ(570–967)	0/37	cytoplasm
Δ(570–776)	11/47	cytoplasm
Δ(785–855)	29/36	cytoplasm
Δ(860–907)	30/41	cytoplasm
GFP-hGli1		
Full length	28/31	cytoplasm
1–527	1/33	cytoplasm/nucleus
Δ(391–655)	0/35	cytoplasm
GFP-hGli3		
Full length	47/69	cytoplasm/nucleus
1–699	0/30	nucleus
Δ(633–1018)	0/30	cytoplasm
GFP-Tra1	0/30	nucleus
GFP-Ci	0/30	cytoplasm

*Cells are considered transfected when nuclear or cytoplasmic GFP signals are present.

We then tested whether the N-terminal region of Gli2 is required for its ciliary localization. Interestingly, we found that the complete removal of the N-terminal region (the first 413 residues) reduces the efficiency of, but does not completely block, the Gli2 ciliary localization (Fig. 1F; Table 1; 8/26 cells exhibit ciliary localization of Gli2). Additional removal of all five zinc-finger DNA binding domains (residues 414–569) further reduces the efficiency of Gli2 localization to the cilia (Fig. 1G; Table 1; 5/69 cells exhibit weak ciliary Gli2 signal).

The above truncation analyses suggest that neither the N-terminus nor the C-terminus is required for the ciliary localization of Gli2, although the N-terminal region apparently contributes to the efficient ciliary localization of Gli2. We subsequently tested whether simultaneous truncation of both ends can completely block Gli2 ciliary localization. Removing 66 residues from the N-terminus and 483 residues from the C-terminus has little effect on Gli2 ciliary localization (Fig. 1H; Table 1; 30/40 cells exhibits ciliary localization). However, two Gli2 variants with more extensive truncations from both ends, Gli2 (414–1061) (n = 0/51) and Gli2 (570–1061) (n = 0/30), fail to localize to the tips of cilia (Fig. 1I and J; Table 1). The lack of ciliary localization is not a result of increased protein degradation because most truncated Gli2 variants are expressed at higher levels than the wild type protein (Fig. S1A–C).

Besides the tips of cilia, the full length GFP-Gli2 protein is also predominantly localized to the cytoplasm, whereas the deletion of the N-terminal domain or part of the C-terminal domain leads to the nuclear accumulation of Gli2 (Fig. 1B–J; Table 1). The nuclear localization of the truncated Gli2 variants does not appear to correlate with the absence of their ciliary localization. For example, Gli2 (1–967) and Gli2 (67–1061) are both localized to the nucleus as well as the tip of the cilium (Fig. 1D, H and Table 1).

### A central region of Gli2 protein is essential for its ciliary localization

We next examined whether the region lying immediately C-terminal to the zinc-finger domains is essential for the ciliary localization of Gli2. We first generated Gli2Δ(570–967), by removing residues 570 to 967, and found that this deletion completely abolished ciliary localization of Gli2 (Fig. 1K; Table 1; n = 0/37). To further define the region essential for Gli2 ciliary localization, we tested three more Gli2 variants with smaller deletions. We found that deletion of residues 570 to 776 drastically decreases, but does not abolish, the Gli2 ciliary localization (Fig. 1L; Table 1; GFP signals are detected in 11/47 cells). In contrast, deletions of residues 785 to 855 (Fig. 1M; Table 1; n = 29/36), or residues 860–907 (Fig. 1N; Table 1; n = 30/41), do not significantly reduce the localization of Gli2 to the cilia. None of these internal deletions leads to the nuclear localization of the Gli2 protein (Fig. 1K–N).

In summary, our deletion analysis identified two important regions in the Gli2 protein that are important for its localization to the tips of cilia. A central region immediately C-terminal to the zinc-finger domains is essential for the ciliary localization of the Gli2 protein. The N-terminal region of Gli2 also plays an important, but not essential role in Gli2 ciliary localization. Immunoblot analyses showed that all Gli2 variants we generated are expressed as predicted ().

### Ciliary localization of mammalian Gli1 and Gli3 requires the central domain

**Figure 2:**
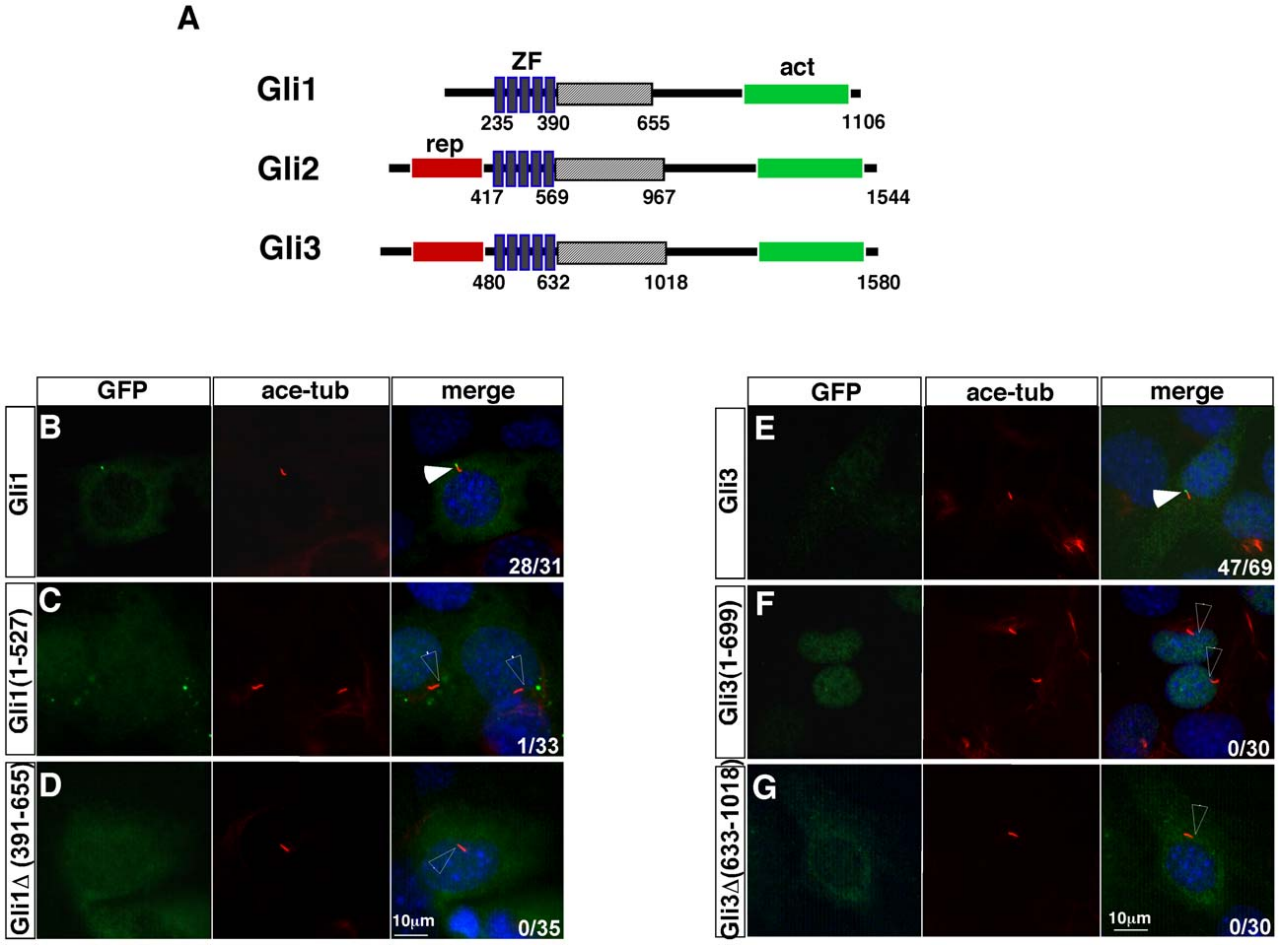
The central regions of Gli1 and Gli3 are essential for their ciliary localization. (A) Schematic illustrations of the three Gli proteins. The repressor domains (rep: red box) are present in Gli2 and Gli3, but not Gli1, although the activator domains (act: green box) are present in all three Gli proteins. The central regions immediately C-terminal to the zinc fingers (ZF) are shown as striped boxes. (B) GFP-Gli1 is localized to the tips of cilia when over-expressed in the ciliated mouse MEFs. (C) Gli1(1–527) is not localized to the cilia. (D) GFP-Gli1Δ(391–655) is not localized to the cilia. (E) Gli3 is localized to the tips of cilia. (F) Gli3 (1–699) is not localized to the cilia. (G) Gli3Δ(633–1018) is not localized to the cilia. Immunofluorescent images of MEFs transfected with GFP-tagged Gli1 and Gli3 variants are shown. Cilia are labeled with acetylated tubulin and nuclei are stained with DAPI. In the merged images, filled arrowheads indicate GFP-Gli proteins at the tips of the cilia, unfilled arrowheads indicate the tips of cilia without GFP-Gli protein accumulation. Numbers at the lower-right corners of each image indicate numbers of cells with ciliary localization of GFP-tagged proteins over total numbers of transfected cells.

All three mammalian Gli family member proteins are localized to the tips of cilia [4]. We examined whether the same mechanism underlying Gli2 localization also regulates the ciliary localization of Gli1 and Gli3. We found that full-length GFP-Gli1 is localized to the tips of cilia in addition to the cytoplasm (Fig. 2A, B; Table 1; n = 28/31). In contrast, Gli1 (1–527), equivalent to Gli2 (1–646), is not localized to the cilia (Fig. 2A, C; Table 1; n = 1/33). In addition, this C-terminally truncated form of Gli1 appears to be in both cytoplasm and nucleus. The deletion of the region immediately C-terminal to the zinc-fingers (residues 391–655) similarly abolishes Gli1 ciliary localization, but does not appear to affect its cytoplasmic localization (Fig. 2A, D; Table 1; n = 0/35).

Over-expressed full-length Gli3 is localized to the tips of cilia, the cytoplasm and the nucleus (Fig. 2A, E; Table 1; n = 47/69). A C-terminally truncated Gli3 protein similar to the processed Gli3 repressor, Gli3 (1–699), fails to be localized to the tips of cilia (Fig. 2A, F; Table 1; n = 0/30). In addition, Gli3 (1–699) is also predominantly accumulated in the nucleus (Fig. 2F and Table 1). Removing the central region immediately C-terminal to the zinc-finger (residues 633–1018) abolishes Gli3 ciliary-localization and decreases its level in the nucleus, similar to what we have observed for Gli1 and Gli2 (Fig. 2A, G; Table 1; n = 0/30). The requirement for a conserved central region for the ciliary localization of all three mammalian Gli proteins suggests a conserved molecular mechanism for targeting these proteins to the cilia.

### Invertebrate Gli homologues are not localized to the cilia when expressed in mammalian cells

**Figure 3:**
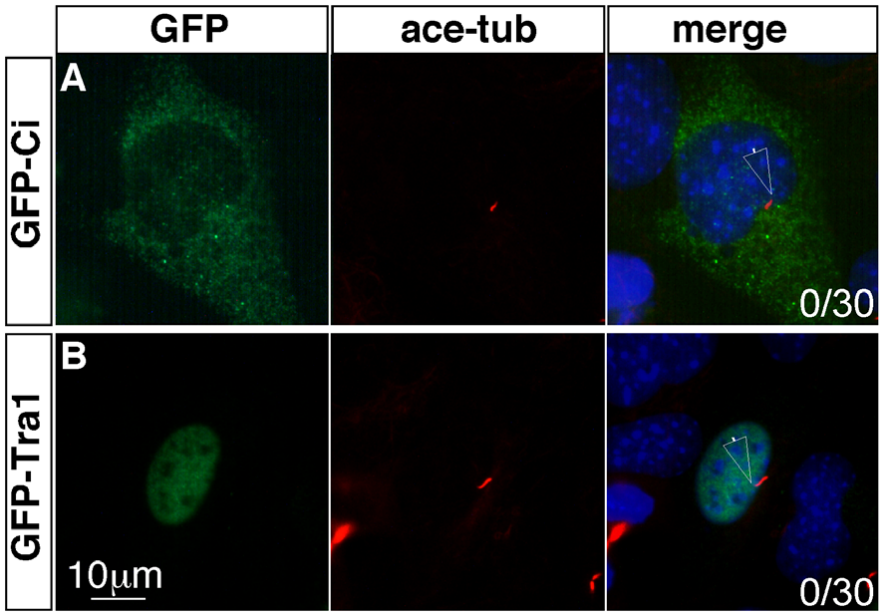
Invertebrate Gli homologues are not localized to cilia upon their introduction into vertebrate cells. (A) GFP-Ci is localized to the cytoplasm when over-expressed in the ciliated mouse MEFs. (B) GFP-Tra1 is localized to the nuclei when over-expressed in the ciliated mouse MEFs. Neither Ci nor Tra1 is localized to the tips of cilia. Immunofluorescent images of MEFs transfected with GFP-tagged proteins are shown. Cilia are labeled with acetylated tubulin and nuclei are stained with DAPI. In the merged images, filled arrowheads indicate GFP signals at the tips of the cilia, unfilled arrowheads indicate the tips of cilia without GFP signals. Numbers at the lower-right corners of each image indicate numbers of cells with ciliary localization of GFP-tagged proteins over total numbers of transfected cells.

Although Hh signaling in mammals requires cilia, cilia are not present in most cells and do not play a role in Hh signal transduction in *Drosophila* [34], [35], [36]. This evolutionary divergence may have resulted in protein-protein interactions that are specific to the mammalian Gli proteins, but absent in their *Drosophila* homologue. To determine whether Ci, the *Drosophila* homolog of Gli proteins, has the structural features that allow for ciliary localization, we expressed GFP-Ci in ciliated mouse MEFs. We found that Ci is predominantly localized to the cytoplasm, but not to the cilia, suggesting that it is structurally diverged from vertebrate Gli proteins (Fig. 3A; Table 1; n = 0/30).

To further study the potential for other Gli homologues to be localized to the cilia, we examined the subcellular localization of Tra1, the Gli homolog in nematode *C. elegans*. Some important Hh pathway components such as Smo and Sufu appear to be missing in *C. elegans*, suggesting a more divergent relationship between Tra1 and vertebrate Gli proteins [37]. We found that when expressed in ciliated MEFs, GFP-Tra1 was predominantly localized in the nucleus and did not accumulate in the cilia (Fig. 3B; Table 1; n = 0/30). The failure of Ci and Tra1 to be localized to the cilia when they are expressed in ciliated MEFs suggests that ciliary localization requires structural features specific to vertebrate Gli proteins.

### Protein kinase A negatively regulates ciliary localization of all Gli proteins

**Figure 4:**
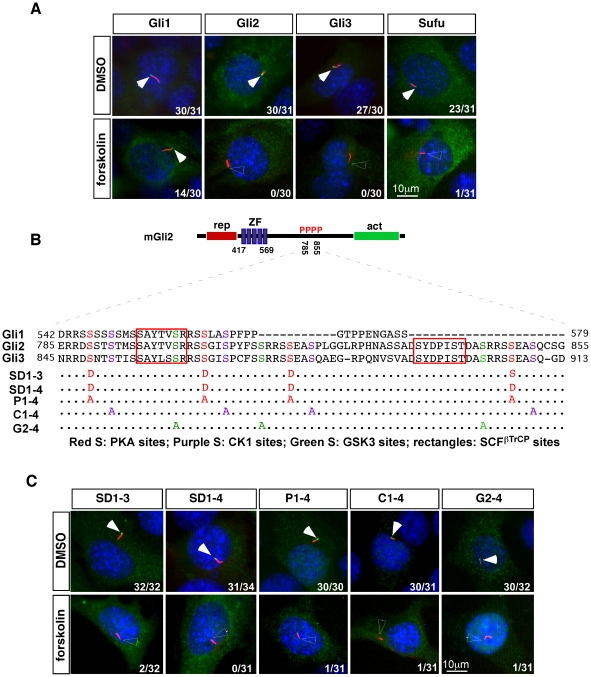
Protein kinase A negatively regulates the ciliary localization of Gli and Sufu proteins. (A) The ciliary localization of GFP-tagged Gli1, Gli2, Gli3 and Sufu are inhibited by a treatment (4 hours for Gli proteins and 18 hours for Sufu) with 40 µM forskolin, but not the solvent (DMSO) alone. The reduction of Gli1 ciliary localization is relatively moderate compared to Gli2 and Gli3 and weak ciliary signal is shown in the image. (B) Peptide sequence of mouse Gli2 protein in the region of residues 785–855, as well as its alignment with Gli1 and Gli3. In SD1-3 and SD1-4, the first three or all four Serine residues targeted by PKA are mutated to Aspartic acids. In P1-4, C1-4 and G2-4, Serine-to-Alanine mutations are created for all PKA, CK1 and GSK3 target sites, respectively. (C) Variants of GFP-Gli2 with mutations in their kinase sites are localized to the cilia in the presence of the solvent (DMSO), but not 40 µM forskolin (FSK) (4 hours of treatment for SD1-3, SD1-4 and P1-4; 18 hours of treatment for C1-4 and G2-4). Immunofluorescent images of MEFs transfected with GFP-tagged Gli proteins are shown. Cilia are labeled with acetylated tubulin and nuclei are stained with DAPI. Filled arrowheads indicate GFP signal at the tips of the cilia, unfilled arrowheads indicate the tips of cilia without GFP signal. Numbers at the lower-right corners of each image indicate numbers of cells with ciliary localization of GFP-tagged proteins over total numbers of transfected cells.

A recent study suggested that protein kinase A (PKA) negatively regulates the ciliary localization of Gli3 [16]. We confirmed that activating PKA with a small molecule agonist forskolin for 4 hours or 18 hours completely abolishes the ciliary localization of full-length Gli3 (Fig. 4A, Table 2, n = 0/30 for 4 hr treatment and 1/31 for 18 hr treatment). We further examined whether PKA activation similarly regulates the ciliary localization of Gli1 and Gli2. Interestingly, we found that forskolin treatment greatly diminishes the ciliary localization of Gli2 (Fig. 4A, Table 2, n = 0/60 for 4 hr treatment and 10/95 for 18 hr treatment), but only moderately reduces the ciliary localization of Gli1 (Fig. 4A, Table 2, n = 14/30 for 4 hr treatment and 26/43 for 18 hr treatment).

**Table 2:** The effects of PKA mediated phosphorylation on the ciliary localization of Gli and Sufu proteins.

Over-expressed proteins	Cells with ciliary localization of the protein of interest/transfected ciliated cells*				
	DMSO		Forskolin		Forskolin plus MG132 (4 hr)
	4 hr	18 hr	4 hr	18 hr	
GFP-Gli1	30/31	30/31	14/30	26/43	—
GFP-Gli2	30/31	86/92	0/60	10/95	0/40
GFP-Gli3	27/30	32/42	0/30	1/31	—
GFP-Sufu	—	23/31	—	1/31	—
Gli2SD1-3	32/32	—	2/32	—	0/32
Gli2SD1-4	31/34	—	0/31	—	0/35
GFP-mGli2P1-4	30/30	30/33	1/31	11/68	—
GFP-mGli2C1-4	—	30/31	—	1/31	—
GFP-mGli2G2-4	—	30/32	—	1/31	—
Gli2Δ(785-855)	—	30/31	—	1/32	—

*Cells are considered transfected when nuclear or cytoplasmic GFP signals are present.

The absence of Gli proteins in the cilia is unlikely the result of increased protein degradation in forskolin-treated cells. First, the levels of GFP-Gli proteins are not grossly affected by forskolin treatment, indicated by both normal GFP signal in the cytoplasm (Fig. 4A), as well as by immunoblot analyses (Fig. S3A). Furthermore, blocking proteasome-mediated protein degradation with MG132 does not rescue the ciliary localization of Gli2 in the presence of forskolin (Table 2). Our immunoblot analysis also indicates that the lack of ciliary localization of Gli2 and Gli3 in forskolin-treated cells is not the result of increased proteolytic processing because the over-expressed Gli2 and Gli3 are both present predominantly in their full-length forms (Fig. S3A). This is further supported by the fact that the ciliary localization of Gli2 variants resistant to proteolytic processing is also efficiently inhibited by forskolin treatment (Fig. 4C, see below).

### PKA-mediated phosphorylation of Gli2 does not appear to play a direct role in its ciliary localization

Based on the fact that PKA activation prevents ciliary localization of Gli3, it was proposed that the phosphorylation of Gli3 by PKA prevents its ciliary accumulation [16]. This model is particularly attractive because the phosphorylation of four Serine residues in a ∼70 amino acid stretch in Gli2 and Gli3 by PKA has been shown to be critical for their proteolytic processing and degradation, likely by priming them for further phosphorylation by CK1 and GSK3β and subsequent association of these two proteins with SCF^βTrCP^ (Fig. 4B) [38], [39]. We thus addressed whether the phosphorylation of these four Serine residues by PKA leads to the lack of ciliary localization of Gli2.

We first examined whether the phosphorylation of Gli2 by PKA is sufficient to inhibit Gli2 ciliary localization. We constructed a series of phosphomimetic forms of Gli2 and examined the localization of two, Gli2SD1-3 and Gli2SD1-4 (Fig. 4B). Strikingly, we found that both Gli2 variants are localized to the cilia (Fig. 4C and Table 2; n = 32/32 for Gli2SD1-3 and 31/34 for Gli2SD1-4). Furthermore, forskolin treatment can greatly reduce the ciliary localization of these two proteins (Fig. 4C, Table 2, n = 2/32 for Gli2SD1-3 and 0/31 for Gli2SD1-4). The decreased ciliary localization of these two proteins is not due to increased protein degradation because addition of MG132 does not rescue their ciliary localization (Table 2 and Fig. S3B; n = 0/32 for Gli2SD1-3 and 0/35 for Gli2SD1-4).

Next we examined whether the phosphorylation of Gli2 by PKA is required for the decrease of Gli2 ciliary localization. In Gli2P1-4, the four Serine residues targeted by PKA phosphorylation are replaced with Alanine; therefore, it can no longer be phosphorylated by PKA (Fig. 4B). Surprisingly, we found that Gli2P1-4 is localized to the tips of cilia in the presence of DMSO (Fig. 4C, Table 2; n = 30/30 for 4 hr treatment and 30/33 for 18 hr treatment), but not in the presence of forskolin (Fig. 4C, Table 2; n = 1/31 for 4 hr treatment and 11/68 for 18 hr treatment). Gli2C1-4 and Gli2G2-4, in which Serines in all target sites for CK1 and GSK3β are mutated to Alanines, respectively, are also localized to the tips of cilia (Fig. 4B, C and Table 2; n = 30/31 for Gli2C1-4 and 30/32 for Gli2G2-4). Forskolin treatment similarly decreased the ciliary localization of these two Gli2 variants (Fig. 4B, C and Table 2; n = 1/31 for both Gli2C1-4 and Gli2G2-4). Consistent with these findings, the ciliary localization of Gli2Δ(785–855), in which all the target sites for the three kinases and SCF^βTrCP^ binding are deleted, is also affected by PKA activation (Table 2; n = 1/32 after 18 hr treatment with forskolin).

In summary, although PKA directly phosphorylates multiple serine residues on Gli2, the phosphorylation of four such residues is neither required, nor sufficient, for the inhibition of Gli2 ciliary localization. The fact that PKA activation can prevent ciliary trafficking of both phosphomimetic and non-phosphorylatable forms of Gli2 suggests that either additional PKA target sites exist in Gli2 and are critically important for its ciliary localization, or PKA regulates Gli ciliary localization indirectly through the phosphorylation of other molecules.

### Gli proteins play critical roles in Sufu ciliary translocation

Sufu is an essential negative regulator of Hh signaling in mammals [17], [18]. Sufu physically interacts with and represses the transcriptional activities of Gli proteins in the absence of cilia, raising the possibility that these proteins may form a complex prior to their ciliary localization [8], [9]. Although previous studies suggested an important role of Sufu in sequestering Ci/Gli proteins in the cytoplasm, it is not required for the ciliary localization of Gli proteins ([9]; and data not shown). Interestingly, a recent report suggested that in *Drosophila*, Ci is essential for the nuclear import of Sufu in the presence of Hh [26]. We therefore examined whether Gli proteins play an important role in the ciliary localization of Sufu.

**Figure 5:**
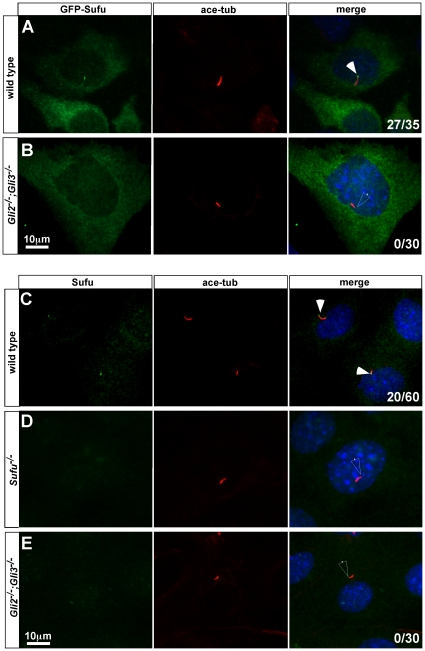
Sufu ciliary localization depends on Gli proteins. (A, B) GFP-Sufu is localized to the cilia in wild type (A), but not in *Gli* mutant cells. (C–E) Endogenous Sufu is localized to the tips of cilia in wild type (C), but not in *Gli2;Gli3* double mutant cells (E). The specificity of the Sufu antibody is confirmed by the loss of Sufu signal in the cilia of *Sufu* mutant MEFs (D). The localization of over-expressed GFP-Sufu is visualized with an anti-GFP antibody (green in A, B), whereas endogenous Sufu is visualized with an anti-Sufu antibody (green in C–E). Cilia are labeled with acetylated tubulin (red) and nuclei are stained with DAPI. In the merged images, filled arrowheads indicate GFP or Sufu signal at the tips of the cilia, unfilled arrowheads indicate the tips of cilia without GFP-Sufu or Sufu accumulation. Numbers at the lower-right corners of each image indicate numbers of cells with ciliary localization of GFP-tagged proteins over total numbers of transfected cells (A, B), or the number of cells with ciliary localization of Sufu over total number of ciliated cells (C, E).

To address the roles of Gli proteins in Sufu ciliary localization, we transiently expressed GFP-Sufu in MEFs derived from *Gli2;Gli3* double mutant mouse embryos. A recent report suggested that *Gli1* is not transcribed in these cells, essentially making them *Gli1;Gli2;Gli3* triple mutants [40]. We will refer to these cells as *Gli* mutant cells. As reported previously, GFP-Sufu accumulated at the tips of cilia in wild type MEFs, in addition to its predominantly cytoplasmic localization ([4]; Fig. 5A and Table 3; n = 27/35 cells). In contrast, GFP-Sufu never accumulates at the tips of cilia in *Gli* mutant cells, even when it is expressed at a high level as indicated by strong cytoplasmic signals (Fig. 5B and Table 3; n = 0/30 cells).

**Table 3:** Subcellular localization of over-expressed and endogenous Sufu in ciliated fibroblasts.

Proteins examined	cells	Cells with ciliary localization of Sufu/total transfected cells*	Sufu localization outside the cilia
GFP-Sufu	Wild type	27/35	cytoplasmic
GFP-Sufu	Gli mutant	0/30	cytoplasmic
Endogenous Sufu	Wild type	20/60**	ND***
Endogenous Sufu	Gli mutant	0/30**	ND***
Endogenous Sufu	Gli mutant plus GFP-Gli1	14/30	cytoplasmic
Endogenous Sufu	Gli mutant plus GFP-Gli2	13/30	cytoplasmic
Endogenous Sufu	Gli mutant plus GFP-Gli2Δ(570–967)	0/30	cytoplasmic
Endogenous Sufu	Gli mutant plus Gli2(1–646)	0/30	cytoplasmic

*Cells are considered transfected when nuclear or cytoplasmic GFP signals are present.

**In these two experiments, all ciliated cells are counted toward the total.

***No obvious signal was detected outside cilia for endogenous Sufu.

We next examined the localization of endogenous Sufu in wild type and *Gli* mutant cells. Using a Sufu-specific antibody, we detected ciliary localization of endogenous Sufu in wild type (Fig. 5C and Table 3; n = 20/60 cells. The lower percentage of cells exhibiting ciliary-localization of Sufu is likely due to the detection limit of the antibody), but not in *Sufu* mutant cells (Fig. 5D), indicating that the staining is highly specific. Interestingly, we observed no ciliary accumulation of Sufu in *Gli* mutant cells, suggesting that Gli proteins are essential for the ciliary localization of endogenous Sufu (Fig. 5E and Table 3; n = 0/30). The lack of ciliary localization of Sufu is not due to a decrease in Sufu protein level because immunoblot analysis indicates no significant decrease of Sufu in *Gli* mutant cells compared to the wild type cells (Fig. S4).

The dependence of Sufu ciliary localization on Gli proteins suggests that PKA activation may similarly inhibit Sufu ciliary localization. Indeed, in the presence of forskolin, the ciliary localization of GFP-Sufu is inhibited (Fig. 4A, Table 2; n = 1/31).

**Figure 6:**
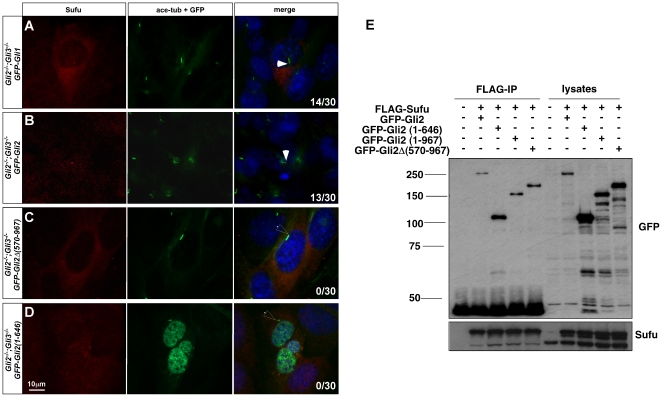
Only ciliary-localized Gli proteins can rescue Sufu ciliary localization. Over-expression of Gli1 (A) and Gli2 (B) rescues ciliary localization of endogenous Sufu. In contrast, over-expression of Gli2Δ(570–967) (C) and Gli2 (1–646) (D), two Gli variants that are not localized to the cilia, fails to rescue the ciliary localization of Sufu. The localization of endogenous Sufu is visualized with an anti-Sufu antibody (red). The over-expressed Gli proteins are visualized through GFP fluorescent signals (green). Cilia are labeled with acetylated tubulin (green) and nuclei are stained with DAPI. In the merged image, filled arrowheads indicate Sufu at the tips of the cilia, unfilled arrowheads indicate the tips of cilia without Sufu accumulation. (E) A co-immunoprecipitation analysis shows that all four Gli2 protein variants physically interact with Sufu. Lysate of cells transfected with FLAG-Sufu and GFP-tagged Gli2 variants was immunoprecipitated with a FLAG antibody and blotted with GFP and Sufu antibodies.

To confirm a direct role for Gli proteins in ciliary localization of Sufu, we expressed either *GFP-Gli1* or *GFP-Gli2* in *Gli* mutant cells. We found that the expression of either gene efficiently restores the ciliary localization of endogenous Sufu in *Gli* mutant cells (Fig. 6A, B and Table 3; only cells exhibiting Gli expression are counted. 14/30 cells expressing Gli1 and 13/30 cells expressing Gli2 show cilia-localization of Sufu). Two Gli2 variants, Gli2 (1–646) and Gli2Δ(570–967), interact with Sufu but are not localized to the tips of cilia (Fig. 1E, K and Fig. 6E). We found that expression of these non-ciliary variants of Gli2 fails to restore Sufu ciliary localization, suggesting that ciliary localization of Gli proteins is a prerequisite for the ciliary localization of Sufu (Fig. 6C, D and Table 3; 30 cells were counted for both variants, none showed cilia-localization of Sufu).

## Discussion

Primary cilia play important roles in Hh signaling and Gli protein activation [41]. Here, we investigated the mechanisms underlying the ciliary localization of mammalian Gli and Sufu proteins. First, we identified a universal requirement for a central region immediately C-terminal to the zinc fingers for the ciliary localization of all three mammalian Gli proteins. Consistent with the evolutionary divergence in regulation of Hh signaling between vertebrates and invertebrates, we found that *Drosophila* and *C. elegans* Gli homologues are not localized to the cilia when expressed in ciliated mouse fibroblasts. We further show that activation of PKA inhibits the ciliary localization of Gli2, Gli3, and to a lesser degree, Gli1. Using Gli2 variants mimicking the phosphorylated and non-phosphorylated forms of Gli2, respectively, we found that direct phosphorylation of Gli2 at four target sites for PKA is not responsible for the lack of Gli2 ciliary localization upon PKA activation. This suggests that PKA may regulate Gli2 ciliary localization through additional target sites on Gli2 or indirectly through the phosphorylation of other proteins. Finally, we show that the ciliary localization of Sufu is dependent on the presence and the ciliary localization of Gli proteins, and is inhibited by PKA activation, suggesting a coordinated ciliary translocation mechanism for Sufu and Gli family of proteins.

### The molecular mechanism of Gli ciliary translocation

All three members of mammalian Gli family are localized to the tips of primary cilia, and at least in the case of Gli2 and Gli3, their ciliary accumulation appears to be regulated by Hh signaling [4], [15], [16]. How this dynamic ciliary localization is regulated at the molecular level remains a mystery. Our data suggest that the central region immediately downstream of the DNA binding domains (residues 391–655 in Gli1; 570–967 in Gli2; and 633–1018 in Gli3) is critically important for the ciliary localization of all of these mammalian Gli proteins. Interestingly, neither the entire N-terminus nor the ∼600 amino acid region at the C-terminus of Gli2 is essential for the ciliary localization; however, loss of both prevents the ciliary localization of Gli2. It is possible that the proper folding of the central region depends on the presence of at least one terminal region. Alternatively, the two terminal regions may play redundant roles in mediating the interaction between Gli2 and a factor important for Gli2 localization. A systematic screen for the Gli interacting proteins and X-ray based structural analysis will be helpful to distinguish between these two possibilities.

It is interesting that the repressor forms of Gli2 and Gli3 are not accumulated in the cilia, despite the requirement of cilia for the efficient processing of Gli3 [4], [5], [6], [7]. It is possible that once full-length Gli proteins arrive at the tips of cilia, they are processed into repressor forms, which exit cilia because of the lack of a mechanism for their retention in the cilia. It was also suggested recently that phosphorylated Gli3 may exit cilia prior to its processing [16]. In either model, Gli repressors may bear modifications acquired while they were inside the cilia that are important for their full activities. Such a scenario would probably be important for our understanding of surprising symptoms of some human diseases. For example, Pallister-Hall Syndrome (PHS) results from a C-terminal truncation of Gli3 protein that renders it a constitutive repressor [42]. Surprisingly, PHS patients and a mouse model of PHS show polydactyly, seemingly inconsistent with a traditional view that Gli3 repressor limits the number of digits in the limbs [43]. A recent report suggested that digit formation may not be directly correlated with the levels of Gli3 repressor activity [44]. Based on our result that Gli3 (1–699), the PHS mutant form of Gli3, fails to be localized to the tips of cilia, we speculate that polydactyly in PHS patients and mutant mice may result from insufficient activation of the Gli3 repressor activity in some context.

It was recently suggested that Kif7, a mammalian orthologue of Cos2, mediates ciliary translocation of Gli3 through direct interaction with the N-terminal region of Gli3 [45]. Our results show that the C-terminally truncated Gli proteins which retain the N-terminal region, Gli1 (1–527), Gli2 (1–646) and Gli3 (1–699), fail to be localized to the cilia, suggesting that interaction with Kif7 is not sufficient for Gli ciliary localization.

The Hh pathway plays pivotal roles in the development of both vertebrate and invertebrate animals, and previous studies have shown conserved roles for many pathway components during evolution. For example, human and frog Gli proteins, when introduced into *Drosophila* wing discs, exhibit transcriptional activities [46], [47]. More importantly, the vertebrate Gli proteins undergo proteolytic processing and are under the control of *Drosophila* pathway components (Hh, Fu, etc), suggesting that vertebrate Gli proteins contain the domains that mediate physical interactions with *Drosophila* Hh pathway components. In contrast, we found that neither *Drosophila* Ci nor nematode Tra-1 accumulates at the tips of cilia when expressed in ciliated mammalian cells, suggesting that additional structural features have evolved in mammalian Gli proteins (or have been lost in fruit flies and nematodes) that allows the interactions that lead to Gli protein localization in the cilia.

### The roles of PKA in the ciliary localization of Gli and Sufu proteins

Our results suggested a negative role for PKA in the ciliary localization of Gli2, Gli3, Sufu, and to a lesser extent, Gli1. Consistent with an important role of PKA in regulating ciliary localization of these critical components of Hh signaling pathway, a recent study found that PKA is localized to the base of cilia [48].

Gli2 and Gli3 are direct targets of PKA, and sequential phosphorylation of four clusters of Serine residues by PKA, CK1 and GSK3 has been shown to be essential for the proteolytic processing of these two proteins [38], [39]. A recent study suggested that PKA prevent ciliary accumulation of Gli3 by direct phosphorylation because only non-phosphorylated Gli3 can be retained in the cilia [16]. To directly address the effects of PKA-mediated phosphorylation of Gli proteins on their ciliary localization, we examined Gli2 variants that mimic either the phosphorylated (Gli2SD1-3 and Gli2SD1-4) or non-phosphorylated (Gli2P1-4; Gli2C1-4 and Gli2G2-4) form of Gli2. To our surprise, both variants mimicking phosphorylated and non-phosphorylated forms of Gli2 are efficiently localized to the cilia and their ciliary localization can both be inhibited by PKA. Consistently, Gli2Δ(785–855), a Gli2 variant missing all these phosphorylation sites plus the binding sites for SCF^βTrCP^, retains its ciliary localization and response to PKA activation. These results suggest that either PKA regulates Gli2 ciliary localization indirectly through the phosphorylation of another protein, or there are additional target sites on Gli2 that can mediate the effect of PKA on Gli2 ciliary localization.

### The coordinated ciliary translocation of Sufu and Gli proteins

Sufu is an essential negative regulator of Hh signaling in mammals [17], [18]. Sufu directly interacts with, and appears to sequester Gli proteins in the cytoplasm [21], [29], [49]. Despite co-localization of Sufu and Gli proteins at the tips of primary cilia, Sufu inhibits Gli activator function in the absence of cilia [4], [8], [9]. The cilia-independent interaction between Sufu and Gli proteins suggests that the assembly of a Sufu-Gli complex occurs prior to their ciliary translocation. Alternatively, this phenomenon can be interpreted as a cilia-independent interaction between Sufu and Gli in the nucleus, although a nuclear role for Sufu has been challenged recently [9], [27], [28], [29]. In the current study, we first showed that Sufu is not localized to the cilia in cells lacking all Gli proteins; second, when PKA activation inhibits the ciliary localization of Gli2 and Gli3, Sufu ciliary localization is similarly inhibited. Finally, re-introduction of ciliary-localized Gli proteins, but not the non-ciliary localized Gli2 variants, can rescue the ciliary localization of Sufu in Gli mutant cells. These results strongly suggest that the ciliary localization of Sufu is dependent on the presence of ciliary-localized Gli proteins and strongly suggest that the assembly of a Sufu-Gli containing protein complex must occur before ciliary translocation. It is interesting that the ciliary-localization of Gli proteins does not require Sufu, ruling out an active role of Sufu in Gli ciliary localization ([9]; and data not shown). We hypothesize that a Sufu-Gli complex is translocated to the cilia through an interaction between Gli proteins and intracellular transport proteins allowing local interactions that relieve the inhibitory effects of Sufu on Gli proteins.

## Supporting Information

Figure S1 **Immunoblots of cells over-expressing GFP-Gli2 variants shown in** **Figure 1** **with an anti-GFP antibody.** (A) Gli2 variants with C-terminal truncation. (B) Gli2 variants with N-terminal truncation. (C) Gli2 variants with truncation at both ends. (D) Gli2 variants with internal deletions. Immunoblots with an anti-tubulin antibody indicate the amount of lysate loaded in each lane. Note that reduced amount of lysate is loaded for some Gli2 variants that are expressed at much higher levels than the full-length Gli2. All lanes are loaded with lysate from cells 24 hours post-transfection unless otherwise indicated. UT: un-transfected control.
(TIF)

Figure S2 **Immunoblots of cells over-expressing GFP tagged Gli1, Gli3, Ci and Tra1 variants shown in** **Figure 2** **and** **Figure 3** **with an anti-GFP antibody.** Immunoblots with an anti-tubulin antibody indicate the amount of lysate loaded in each lane. The *Drosophila* Ci is expressed at a very low level in mouse cells such that it is barely detectable in immunoblots (asterisk), but its expression can be detected in some cells through immunocytochemistry.
(TIF)

Figure S3 **(A) Immunoblots of cells over-expressing GFP-tagged Gli1, Gli2, Gli3 and Sufu (bands indicated by arrowheads) in the presence of solvent (DMSO) or forskolin (FSK).** Forskolin-treatment does not lead to a decrease in the level of these proteins. (B) Immunoblots of cells over-expressing GFP tagged Gli2, Gli2SD1-3 and Gli2SD1-4 in the presence of solvent (DMSO), forskolin, MG132 or forskolin plus MG132. Note that neither forskolin nor MG132 treatment dramatically changes the level of these over-expressed Gli2 variants. Immunoblots with an anti-tubulin antibody indicate the amount of lysate loaded in each lane.
(TIF)

Figure S4 **Immunoblots of wild type,** ***Gli*** **mutant cells and** ***Gli*** **mutant cells transfected with GFP-Gli1 with an anti-Sufu antibody.** Note that the overall levels of endogenous Sufu are comparable between these cells. *Sufu* mutant cells serve as a negative control to show the specificity of the Sufu antibody. Immunoblots with an anti-β-actin antibody indicate the amount of lysate loaded in each lane.
(TIF)
