# Supplementary figures and images for: Coordinated Translocation of Mammalian Gli Proteins and Suppressor of Fused to the Primary Cilium
Source: PLoS One. 2010 Dec 29;5(12):e15900. doi: 10.1371/journal.pone.0015900 (PMC3012114; doi:10.1371/journal.pone.0015900)

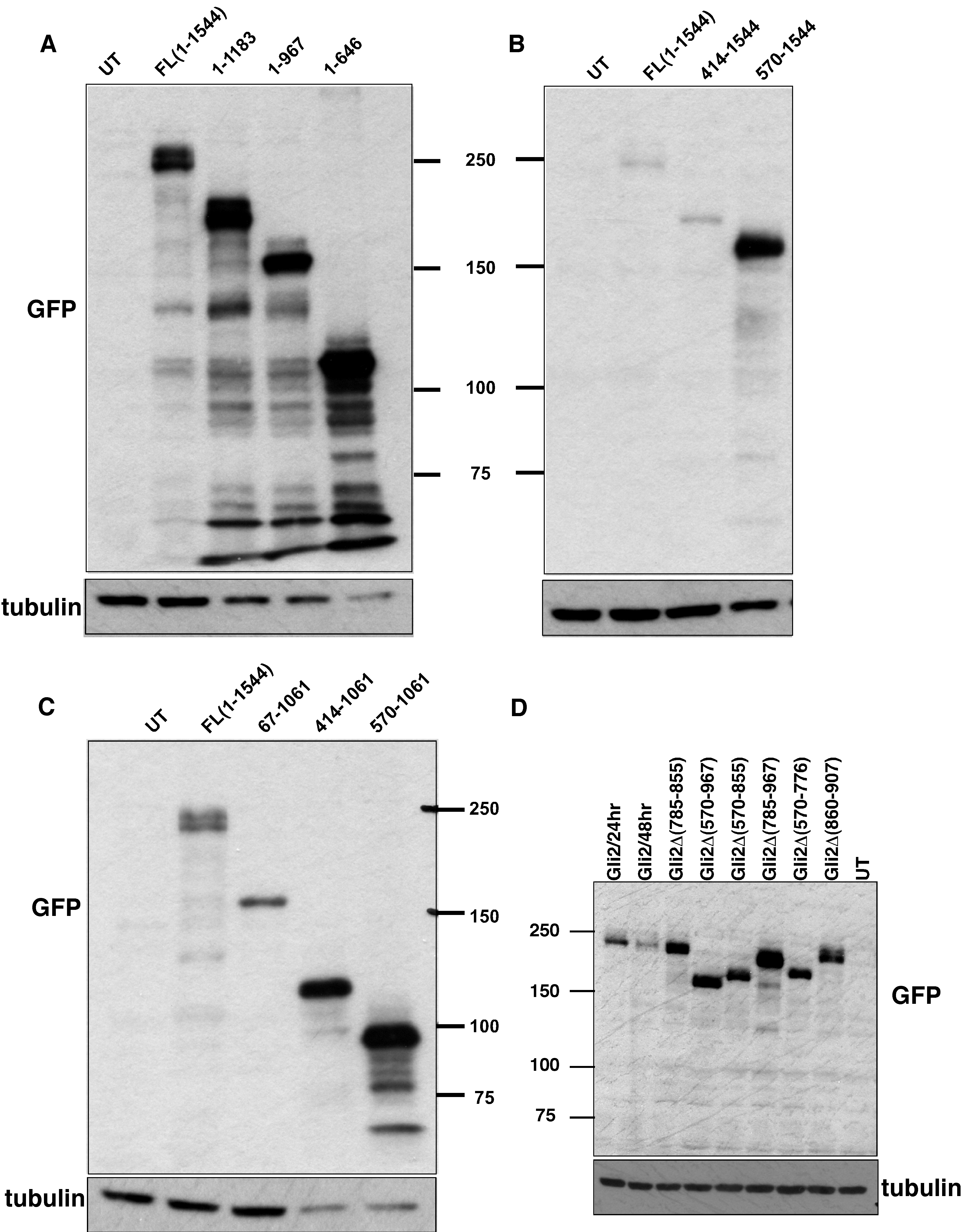

Supplement: Figure S1 — Immunoblots of cells over-expressing GFP-Gli2 variants shown in Figure 1 with an anti-GFP antibody. (A) Gli2 variants with C-terminal truncation. (B) Gli2 variants with N-terminal truncation. (C) Gli2 variants with truncation at both ends. (D) Gli2 variants with internal deletions. Immunoblots with an anti-tubulin antibody indicate the amount of lysate loaded in each lane. Note that reduced amount of lysate is loaded for some Gli2 variants that are expressed at much higher levels than the full-length Gli2. All lanes are loaded with lysate from cells 24 hours post-transfection unless otherwise indicated. UT: un-transfected control. (TIF) [file pone.0015900.s001.tif]

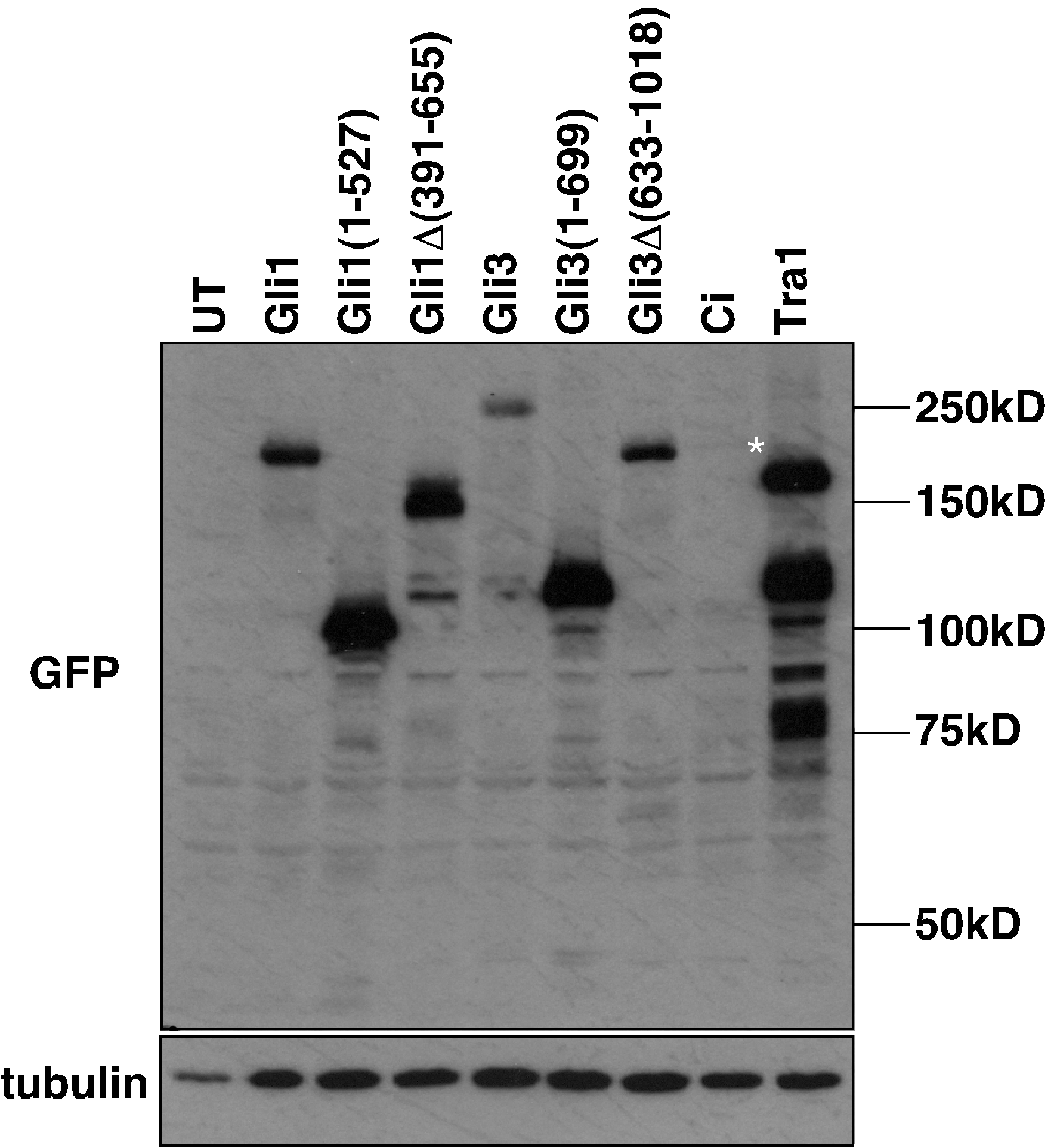

Supplement: Figure S2 — Immunoblots of cells over-expressing GFP tagged Gli1, Gli3, Ci and Tra1 variants shown in Figure 2 and Figure 3 with an anti-GFP antibody. Immunoblots with an anti-tubulin antibody indicate the amount of lysate loaded in each lane. The Drosophila Ci is expressed at a very low level in mouse cells such that it is barely detectable in immunoblots (asterisk), but its expression can be detected in some cells through immunocytochemistry. (TIF) [file pone.0015900.s002.tif]

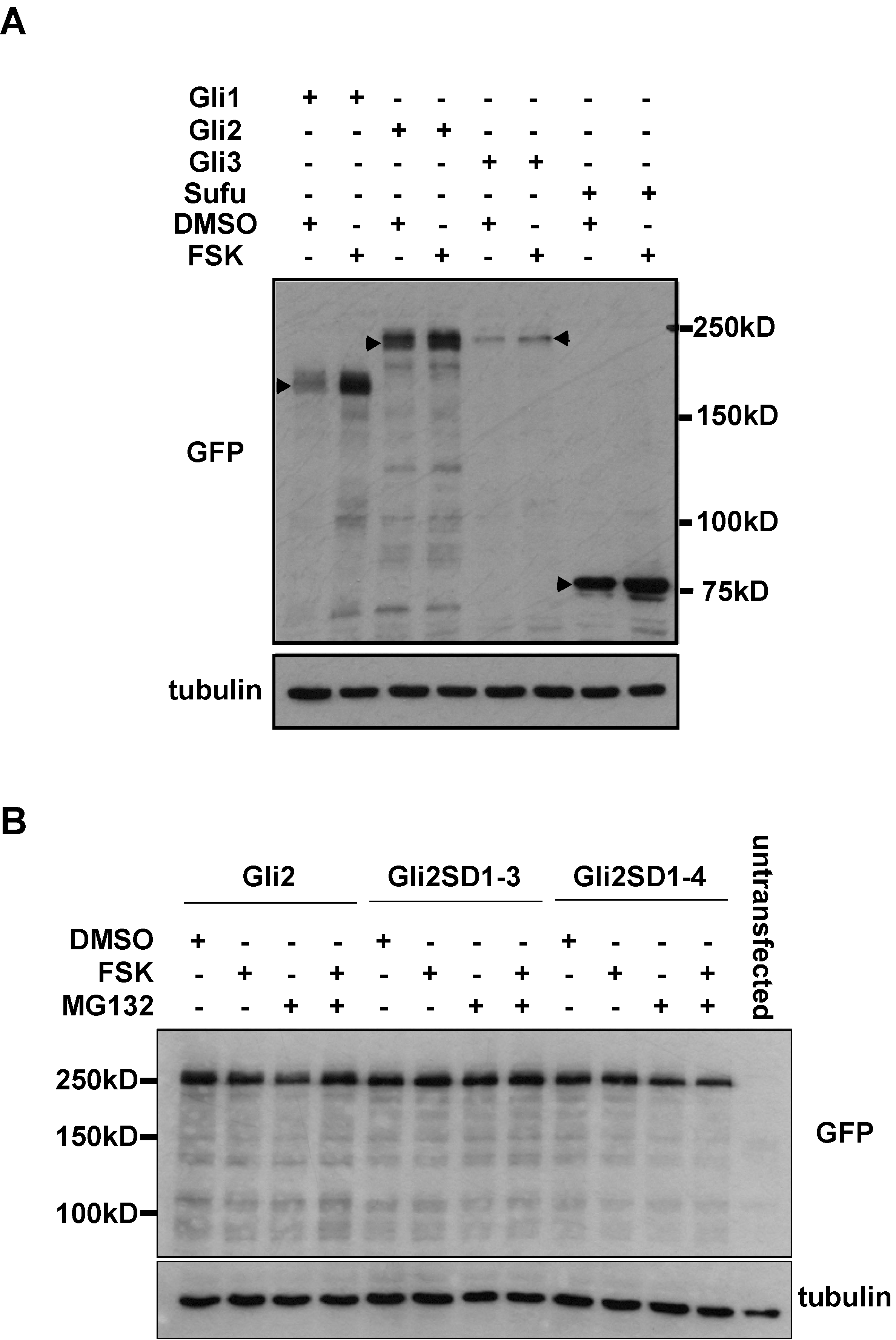

Supplement: Figure S3 — (A) Immunoblots of cells over-expressing GFP-tagged Gli1, Gli2, Gli3 and Sufu (bands indicated by arrowheads) in the presence of solvent (DMSO) or forskolin (FSK). Forskolin-treatment does not lead to a decrease in the level of these proteins. (B) Immunoblots of cells over-expressing GFP tagged Gli2, Gli2SD1-3 and Gli2SD1-4 in the presence of solvent (DMSO), forskolin, MG132 or forskolin plus MG132. Note that neither forskolin nor MG132 treatment dramatically changes the level of these over-expressed Gli2 variants. Immunoblots with an anti-tubulin antibody indicate the amount of lysate loaded in each lane. (TIF) [file pone.0015900.s003.tif]

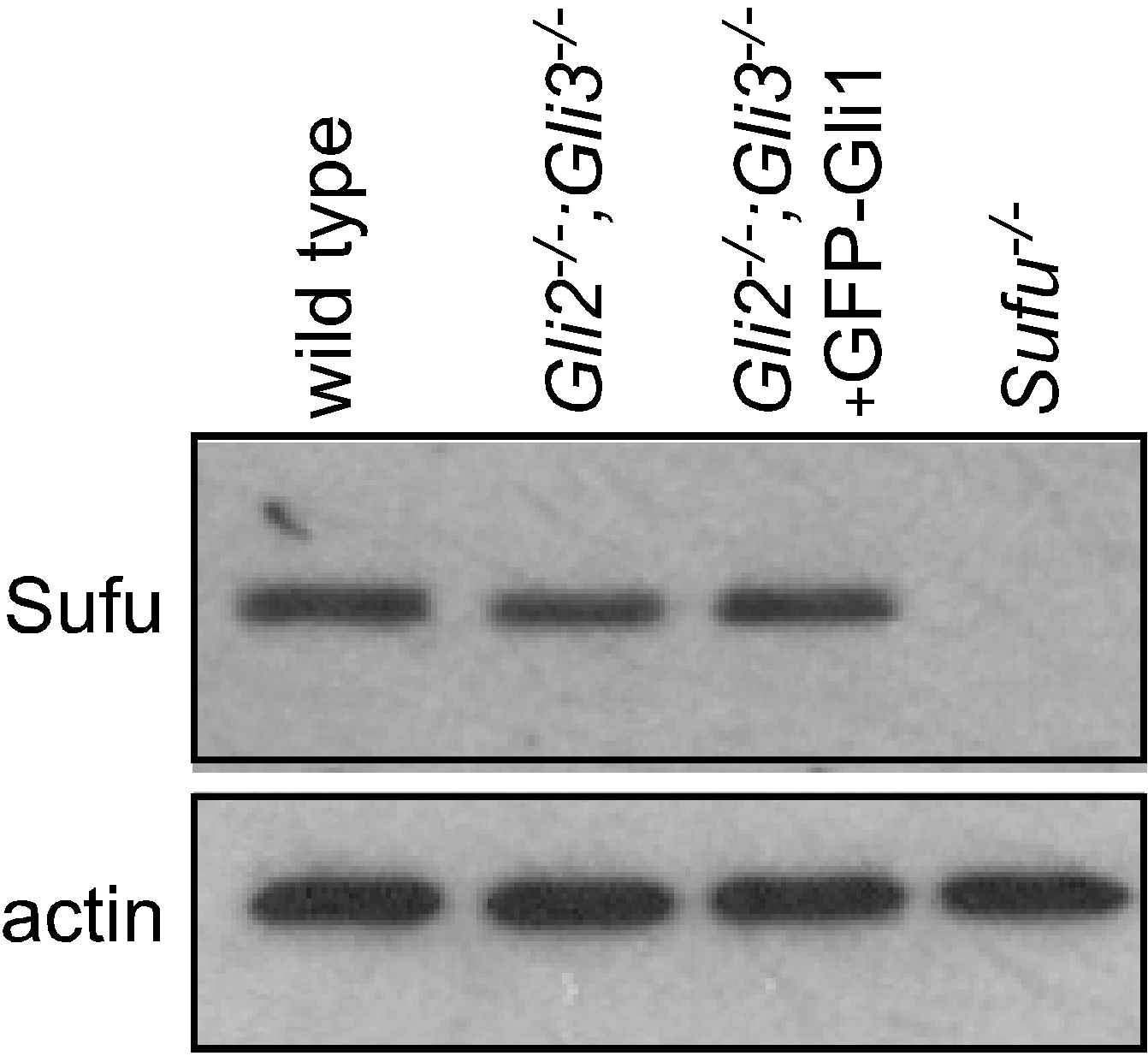

Supplement: Figure S4 — Immunoblots of wild type, Gli mutant cells and Gli mutant cells transfected with GFP-Gli1 with an anti-Sufu antibody. Note that the overall levels of endogenous Sufu are comparable between these cells. Sufu mutant cells serve as a negative control to show the specificity of the Sufu antibody. Immunoblots with an anti-β-actin antibody indicate the amount of lysate loaded in each lane. (TIF) [file pone.0015900.s004.tif]
